# Caught between two worlds: mental health literacy and stigma among bicultural youth

**DOI:** 10.1080/17482631.2024.2321644

**Published:** 2024-03-03

**Authors:** Ariel Kwegyir Tsiboe, Shruthi Raghuraman, Tara C. Marshall

**Affiliations:** a Department of Health, Aging & Society, McMaster University, Hamilton, Canada; b Department of Psychology, Neuroscience, and Behaviour, McMaster University, Hamilton, Canada

**Keywords:** Bicultural youth, acculturation, mental health literacy, culture, stigma, Canada

## Abstract

**Purpose:**

Bicultural youths are at higher risk of mental health problems and are less likely to utilize mental health services, yet our knowledge of their mental health literacy and help-seeking behaviours remains limited.

**Methods:**

To fill this gap, the current study explored bicultural youths’ mental health literacy and stigma by conducting semi-structured interviews with 14 Canadian university students in 2021.

**Results:**

Our analysis revealed that bicultural youths may be torn between two worlds: intergenerational tensions between participants assimilated into individualistic Canadian culture and their more collectivist parents meant that they had different cultural perceptions of mental health literacy and stigma. While being caught between these two worlds may be detrimental for bicultural youth, our results also suggested that a trans-cultural factor—celebrities’ mental health journeys—may promote help-seeking behaviour across participants. Furthermore, our study speaks to the ways that unprecedented events such as the COVID-19 pandemic impact mental health literacy among bicultural youth. Our findings might be used by university mental health services to encourage help-seeking among bicultural students.

**Conclusion:**

The acculturation of mental health literacy, stigma, and associated intergenerational differences needs to be considered by university wellness services.

## Introduction

Canada is becoming an increasingly multicultural country. In 2016, 22% of Canada’s population (i.e., 7.6 million residents) reported being a member of an ethnic group, an estimate which is expected to increase by one-third by 2031 (Statistics Canada, 2017). Asia and Africa have replaced Europe as the largest source region of Canadian immigrants: in 2021, 62% of immigrants were from Asia or the Middle East, 16% were from Africa, and only 10% were from Europe (Statistics Canada, 2022).

Due to this rise in multiculturalism and intercultural contact, an increasing number of Canadians identify as bicultural (Statistics Canada, 2017). Biculturalism refers to the exposure to and internalization of two distinct cultures that may be experienced by migrant, indigenous, and biracial individuals (Nguyen & Benet-Martínez, 2007). There tend to be elevated rates of mental health problems among bicultural individuals, yet there is also an underutilization of mental health services in this population (Baker et al., 2012; Thomson et al., 2015). Bicultural youths may be at particular risk for mental health problems given that the majority of persons who develop mental illness do so between the ages of 16 and 25, an age frame in which many young people embark on their undergraduate education (Almanase, 2021). During this period, many young people often encounter the challenges of integrating two distinct cultural identities which often involve challenging experiences, including family expectations and pressure, discrimination, and social isolation (Crane et al., 2005). Poor mental health can have steep consequences for undergraduates, including higher risk of dropout and lower employability after graduation (Gorczynski & Sims-Schouten, 2022). Bicultural university students tend to have lower rates of diagnosed psychiatric illness compared to their white peers, yet higher rates of mental health symptoms, suggesting that they may delay seeking help after the initial onset of symptoms (Amri & Bemak, 2012). University campuses tend to offer support services in ways that are meant to encourage mental health utilization and literacy (Kutcher et al., 2015), including providing information and access to counselling. However, many students—especially bicultural students—report them as being ineffective due to the lack of cultural sensitivity and awareness of the unique cultural experiences of bicultural youth (Amri & Bemak, 2012). The lack of multicultural understanding among mental health professionals may also contribute to a disconnect, posing challenges for bicultural individuals in feeling adequately supported and understood (Emerson et al., 2021; Hampton & Sharp, 2014). Another reason for this disconnect may be the stigma associated with mental health help-seeking in various cultural communities (Altweck et al., 2015). There may be discrepancies in beliefs about mental health between bicultural youths’ culture of origin and the country they live in (Emerson et al., 2021). This nuanced perspective is poorly understood and there is little research exploring mental health literacy and stigma that could support a deeper understanding of this issue. Thus, students’ mental health may continue to be suboptimal until we know more about the perspectives and experiences of bicultural university students. In this paper, we report the results of a qualitative study that explored the nature of mental health literacy and ways to reduce mental health stigma among bicultural undergraduate students in Canada.

### Mental health literacy

Mental health literacy (MHL) model has been widely studied and promoted in Western societies (Gorczynski & Sims-Schouten, 2022; Gorczynski et al., 2017; Kutcher et al., 2015). It refers to the “knowledge and beliefs about mental health disorders which aid their recognition, management or prevention” (Jorm et al., 1997, p. 184). The MHL model is multifaceted and includes beliefs that promote recognition, knowledge regarding the causes of mental disorders, the ability to recognize symptoms of mental illness, seeking professional help, and seeking lay sources of help. Better knowledge and more positive beliefs about mental illness can pilot pathways to help-seeking and responses to treatment. For example, being able to recognize symptoms of mental illness and providing a psychiatric description or label can initiate a schema about appropriate action that an individual can take, such as seeking professional help, taking medication, or avoiding potentially harmful self-help- strategies such as substance abuse or managing alone (Jorm, 2012; Jorm et al., 2006).

Efforts to understand MHL have received international research attention, especially factors associated with MHL such as stigma and health-seeking behaviours in non-Western populations (Beasley et al., 2020; Furnham & Hamid, 2014). However, very few studies have focused on the MHL of bicultural youth, especially within the student population (Beiser et al., 2011; Cheung et al., 2011; de Moissac et al., 2020). More work needs to fill in the gaps in our understanding of how MHL is informed by culture among bicultural youth.

### MHL and culture

While the MHL framework is consistent with Western culture’s focus on scientific understanding of mental illnesses, it may conflict with some non-Western cultural views of mental health (Jorm, 2012). Below, we discuss several reasons why bicultural youth in Western cultures may experience MHL differently than their monocultural counterparts or have poorer mental health: (a) diverse conceptualizations of mental health across cultures and the role of collectivism, (b) intergenerational differences between parents and bicultural youths, and (c) navigating between two cultures.

Culture shapes many aspects of mental health, ranging from the ways in which various mental illnesses are perceived to pathways of seeking help (Altweck et al., 2015; Kramer et al., 2002). For example, some cultures may ascribe the onset of mental illness to possession by evil spirits and black magic, while others promote healing temples and other shrines for people who experience mental illness (Gopalkrishnan, 2018). Some Chinese migrants may view mental health issues as a manifestation of a lack of balance in one’s mental and spiritual well-being, such that healing often incorporates spiritual and religious traditions involving acupuncture and herbs, whereas others may perceive mild mental illness as personal problems to be addressed by an elder in the family or a spiritual leader (Kramer et al., 2002; Long et al., 2021). Varying conceptualizations of mental illness across cultures often stem from differences in collectivism (Altweck et al., 2015). Collectivism refers to prioritizing the values and ideals of the ingroup over personal goals for the purpose of preserving ingroup harmony, interdependence, security, conformity, and tradition (Schwartz, 1990). Because of the desire to maintain social harmony, save face, and uphold the family reputation in collectivist cultures, people might engage in somatization rather than psychologization of mental health problems (Markus & Kitayama, 1991; Ryder & Chentsova-Dutton, 2012). This means that they might focus on somatic aspects of a mental health complaint like poor appetite or not sleeping instead of psychological symptoms like low mood and lack of motivation—thereby side-stepping any potential mental health stigma. Some individuals may have high levels of MHL but feel reluctant to endorse certain help-seeking behaviours due to their collectivistic cultural values. Thus, the lack of use of mental health resources would be due to cultural beliefs and not necessarily due to low MHL (Samari et al., 2022). This highlights the importance of considering cultural perspectives when addressing mental health literacy and tailoring effective help-seeking behaviours to diverse populations.

In addition to the influence of collectivism, bicultural youths’ MHL and mental health may be affected when parents and their offspring have different acculturation goals and experiences (Shi et al., 2019; Wang et al., 2019). Specifically, parents tend to be less acculturated than their children, a phenomenon that has been referred to as the acculturation gap (Asvant & Malcarne, 2008; Shi et al., 2019). Although bicultural youth of collectivist cultural backgrounds who are socialized in Western cultures tend to be committed to the values and beliefs of their heritage culture, they also tend to be more acculturated to Western cultures than their parents because they experience both cultures at a relatively young age (Kramer et al., 2002). This may result in intergenerational tension that decreases bicultural youths’ MHL and seeking of professional help (Asvant & Malcarne, 2008; Cheung et al., 2011). Alternatively, highly assimilated bicultural individuals may experience *heightened* MHL and well-being insofar as they wish to endorse beliefs—such as mainstream Western culture’s emphasis on MHL—that are in contrast to those of their less-assimilated family members (Amri & Bemak, 2012; Asvant & Malcarne, 2008; Berry, 1990).

Another influence on bicultural youths’ MHL and experience of mental health problems is that they are often torn between two cultures (Long et al., 2021) and experience acculturative stress (Pang et al., 2017). For example, Muslims residing in Western countries share a culture based on the traditions, values and beliefs of the Islamic faith, a hierarchical and extended family structure, and a collectivist approach to social relations, which in many respects differs from the dominant Judaeo-Christian faith, egalitarian and nuclear family structures, and individualistic approach to social relations in Western cultures (Amri & Bemak, 2012; Asvant & Malcarne, 2008). Bicultural youths who have adopted mainstream cultural values alongside maintenance of their heritage culture may successfully negotiate between two cultural frames that vary in their conceptualizations of mental health (Benet-Martinez et al., 2021; Berry, 1990). However, this negotiation may also be contested (Bhatia & Ram, 2001), and the frame-switching difficult (Benet-Martinez et al., 2021). This may result in acculturative stress, which may challenge one’s fundamental values and beliefs (including ideas about mental health and help-seeking) and lead to clinical symptoms such as depression and anxiety (Berry, 1990).

### MHL and stigma

Bicultural youths not only negotiate competing cultural conceptualizations of mental health, but also cultural differences in mental health stigma. Stigma is defined as a “socially constructed mark of disapproval, disgrace or shame that causes significant disadvantage through the limitation of opportunities” (Martin, 2010, p. 261). It includes both public stigma (i.e., treatment of the stigmatized group by a prejudiced public) and self-stigma (i.e., stigmatized individuals’ internalization of public stigma) (Corrigan, 2004). The shame that results from mental health stigma has been associated with negative attitudes towards professional help-seeking and the underutilization of mental health services (e.g., not seeking help at all, seeking help but with low adherence, or ending treatment early; Corrigan, 2004).

One of the key functions of the MHL model is to understand how individuals experiencing mental illness seek appropriate help (Jorm et al., 1997). The incidence of mental health stigma in collectivist cultures may discourage individuals from professional help-seeking (Altweck et al., 2015; Nguyen & Bornheimer, 2014; Pang et al., 2017). Shame resulting from mental health stigma in one’s heritage culture community may be a reason for the low mental health service utilization of acculturating individuals (Amri & Bemak, 2012; Emerson et al., 2021; Hampton & Sharp, 2014). The desire to protect the family reputation and honour in collectivist cultures—e.g., to attract marriage partners for children—may also drive low service utilization (Gopalkrishnan, 2018; Uhlich et al., 2022). Accordingly, families may feel compelled to hide their mental health problems to protect the children and the entire family from the tag “crazy” or other marks of external shame (Hampton & Sharp, 2014). Because people with mental illness in collectivist cultures may be viewed as unpredictable and their illness a sign of personal weakness, they often feel embarrassed if they were diagnosed with mental illness or would not want others to know if they had a mentally ill relative (Pang et al., 2017). Furthermore, cultures that are higher in gender role traditionalism may particularly stigmatize and inhibit men’s expression of mental health problems (Chatmon, 2020). For example, in many African cultures, there’s a notion that “men don’t cry and big boys don’t share tears” (Ezeugwu & Ojedokun, 2020, p. 4). Taken together, mental illness may be seen as a mark of shame among bicultural youths with collectivist cultural backgrounds; it is something they fear their family and peers might stigmatize. This mental health stigma can negatively affect their coping strategies; meanwhile, those who perceive lower cultural stigma are more likely to seek appropriate help, which is seen more in monocultural Westerners than in bicultural Westerners (Samari et al., 2022).

## The present study

There is a lack of research regarding bicultural youths’ lived experiences of MHL and stigma in multicultural countries like Canada. Without rich and contextualized information, it is difficult to develop recommendations and programmes that will encourage this vulnerable group to seek mental health services, despite cultural stigma. The present study adds to the few existing studies by engaging youths from diverse cultural backgrounds rather than focusing on participants of one cultural group, thus allowing us to examine commonalities across cultural backgrounds. Insofar as bicultural students’ mental health help-seeking behaviours continue to be sub-optimal, and campus mental health services ineffective, the present study may add to our knowledge regarding this group’s mental health needs and offer some new directions. Given that quantitative scales and assessments currently dominate the literature regarding MHL and bicultural youth, the aim of the present study was to understand bicultural youths’ MHL, barriers to mental health services, and the way forward through conducting in-depth interviews with a university population in Canada.

## Method

### Study design

We used a phenomenological approach to arrive at a more in-depth understanding of participants’ lived experiences (Bryman, 2016). Our research team consisted of three scholars from a large urban university in Southern Ontario: the first author is Ghanaian, the second author is a Canadian of South Indian heritage, and the third author is a Canadian of British heritage. Ethical clearance was attained from our university’s ethics review board.

### Context

In the 2020/2021 academic year, the authors’ university registered 31,532 undergraduate students, of whom 15.75% were international students from 120 countries (Discover McMaster, 2020). The present study sampled participants who self-identified as bicultural from the university’s undergraduate population.

### Participants

We recruited participants using a purposive sampling technique, which is aimed at identifying diverse participants who share the same experiences of a central phenomenon—in this case, biculturalism. To participate in the study, participants passed eligibility criteria based on (a) age: must be 18 and older, (b) self-identifying as bicultural, (c) open to talking about their mental health and related cultural challenges. We recruited participants by emailing undergraduate students enrolled in the academic programme directed by the third author (who did not liaise with any participants). This yielded five participants per our criteria. Since the study involved sharing sensitive experiences of participants’ lives, many of our potential participants may have been hesitant to participate in the study. This led us to adopt other approaches such as snowball sampling and advertising the study on some of the universities’ social media platforms—approaches that yielded nine more participants. All 14 participants were undergraduate students who came from diverse cultural backgrounds and various academic disciplines. It is important to note that it was difficult to recruit male participants, perhaps at least in part because undergraduate students enrolled in the social sciences at our university are predominantly female. During interviews, we ensured that the data sufficiently addressed our research questions and provided a comprehensive understanding of the phenomena under investigation. After conducting 14 interviews, the interviewer made a judgement that saturation had been reached. Themes were repeating and the last interviews were not yielding new information.

Participants were 86% female-identified with an average age of 19 years (*SD* = .78). Most were in their second year of study at university (71%) and from the Faculty of Social Sciences (57%). The most common heritage culture was Indian (29%), followed by Filipino (21%) and Middle Eastern (14%). The rest identified various South American or European heritage cultures. 64% were born in Canada or moved to Canada at a young age.

### Interviews

Before each interview, participants were provided with information sheets concerning the study, including assurances of confidentiality and anonymity. Each participant then signed a consent form and was reminded that their participation in the study was voluntary. Semi-structured interviews were conducted with each participant in English between July—September 2021. The interview guide began by asking demographic questions such as gender, age, and cultural background. This was followed by open-ended questions that explored participants’ experiences and their understanding of mental health, including the following: What does mental health mean to you? What do you think causes mental illness? What do you think influences individuals’ perceptions of people with mental illness in your culture? How have the cultural attitudes towards mental health shaped your perspective on seeking mental health treatment? Have you ever experienced mental health stigma within your cultural community before? During interview sessions, the use of probes was vital as it enhanced the richness of data (Bryman, 2016). To follow social distancing protocols during the COVID-19 pandemic, interview sessions were held online via Zoom technology and lasted for a maximum of 60 minutes. An automatic verbatim transcription was created by Zoom and the second author edited them for readability. Pseudonyms were given to participants according to the order of the interviews. For example, the fifth person to be interviewed was identified as participant five and the second was denoted as participant two.

A reflexive journal was kept during the interview sessions to mitigate possible biases by prompting greater self-awareness during analysis. For example, the interviewer—the second author, who is female—noted that her age did not seem to influence the interview sessions, but female participants seemed more comfortable in openly sharing their experiences and feelings compared to male participants. Additionally, she observed that many of the participants seemed to feel more comfortable sharing their experiences because she was also from a collectivist cultural background where mental illness is stigmatized. This further allowed her to empathize with their personal experiences during interview sessions, adding to the richness of the data. Having said this, we were careful to not let our ideas, connections, and perceptions about the topic influence interview sessions and our analysis.

### Analysis

All interview transcripts were analysed using reflexive thematic analysis (Braun & Clarke, 2006), facilitated by NVivo 12 software. Transcripts were read and re-read to familiarize ourselves with the data. An open coding approach was adopted, where codes were used to categorize topics and meanings in each transcript without a pre-existing coding list. Codes of similar meanings were merged or renamed as appropriate (Bryman, 2016). Later, codes were moved into clusters that denoted the same concept to generate coding trees. The link between research questions and clusters of codes was explored and tested for robustness by returning to the data fragments in NVivo and the full transcripts to view codes in their original context (Braun & Clarke, 2006). A deeper exploration of our interpretations of participants’ experiences resulted in our various overarching themes. Themes were discarded if they did not have enough evidence to support them. Other themes were strengthened and amended through the process of exploring the data, and discussion with the research team.

The Consolidated Criteria for Reporting Qualitative Research guidelines were followed to ensure quality and rigour (Bryman, 2016). An audit trail was kept which maintained adaptability and confirmability; this included information regarding decision-making during coding (Bryman, 2016). The first and second authors performed independent iterative analysis, arriving at similar themes in the end. Final codes and themes that emerged from the data were further discussed among the research team to ensure validity. Next, we discuss each theme in turn.

## Results

### The development of understanding of mental health

Participants’ understanding of mental illness reflected some of the components of Jorm’s et al. (1997) MHL model (e.g., recognition of and knowledge about mental health). Despite the diversity in participants’ academic programmes, all participants showed a good understanding of mental health and mental illness during interviews. Participants’ understanding encompassed cognitive, social, psychological, environmental, and behavioural aspects of mental health. One psychology student said, “*I think mental health means our emotional and psychological well-being. So, I feel like mental health affects the way we think and feel as well as how we handle stress*” (Participant 2, Chinese Filipino). Meanwhile, a software engineering participant described mental health as, *“Feeling good about yourself and comfortable at a point in life and having a support system around you that will help you through everything you’re going through and basically, just not feeling alone”* (Participant 3, Egyptian).

Participants were also able to demonstrate that they were well-informed about the causes of mental illness, further reflecting elements of Jorm’s et al. (1997) theoretical model of MHL: *“I believe there’s not a single cause of mental illness, it could be either a biological cause for example genetics, or it could also be a psychological cause like childhood trauma or stressful events like losing a parent or losing a loved one. It could also be the social environment in which the person grew up and the fact that the person doesn’t have a positive outlook in life hence, probably a negative thinking cycle”* (Participant 1, Filipino). As mentioned by another, “ *… What around you can cause it [mental health]. Of course, social media too, the internet and how we interact with it because that’s really changed.”* (Participant 12, Kenyan).

This understanding of mental health among our diverse sample was influenced by the continuous exposure to mainstream Canadian culture: “*Like it’s different for me because, as someone who also lived in the Philippines for eleven years and living in Canada now for the past eight or nine years, my experiences with mental health and talking about mental health is different now … . Yeah, most of the influence I have on mental health were learnt in Canada”* (Participant 2, Chinese Filipino).

Having said this, most of the participants did not know much about what mental health and mental illness really meant prior to commencing their undergraduate degrees in Canada. This was often attributed to their cultural and collective socialization which limited exposure to mental health issues before university. For example, one participant reported that mental health issues were not discussed in their home: *“In terms of being a Muslim, it’s very conservative and it [mental health] is not treated properly at all”* (Participant 8, Indian). Another said, “*My family is very open where we discuss our problems … but normally there was no mention of mental health or if I was feeling stressed with my studies or felt that I was been overwhelmed with something which I didn’t know what to do, most of the time I chose not to speak up*” (Participant 8, Indian). A few participants discussed the lack of a word in their local dialects that denotes mental health. These participants substituted “mental illness” with stigmatizing words such as “crazy” and “mad”: *“There isn’t really a word for mental illness in my cultural community … When someone is mentally ill, they call him crazy or something*” (Participant 2, Chinese Filipino).

Conversely, having educated and urban parents from India exposed one participant to issues concerning mental health: *“For me, my parents are highly educated. My mother is a doctor, and my dad is an engineer, and because of this they can communicate and share ideas at a higher level and bring forth real issues. Because of that, my parents are more progressive and more open. Although they still have some form of conservative side, I was still able to grow up to be a bit more open than I would say for other people who are in the same situation [culture] as me”* (Participant 1, Filipino).

The COVID-19 pandemic was only mentioned once throughout all 14 transcripts. It led one conservative family to suddenly open up to discuss mental health issues due to the fear and panic the pandemic brought: *“It [mental health] was never discussed. I feel like my family only started discussing it now during the pandemic … . So, during the lockdowns, there were seven people driving each other crazy in the house every day and you can’t leave. That was when my mental health started going down … . so that was when we actually started talking about it [mental health]”* (Participant 1, Filipino).

### Cultural perceptions of mental health

When participants reported on their perceptions regarding mental health as experienced in mainstream Canada and in their various heritage countries, they often seemed caught between two worlds. All participants reported that they were able to more freely discuss issues concerning mental health in Canada compared to their heritage culture: *“In Canada, people are more open, and they speak about their experiences [mental health]. I feel like if I tell someone that I think I might have a problem and I will need to go to a therapist, people will be encouraging and supportive of me but not in Ukraine”* (Participant 13, Ukrainian).

Often, participants felt that their heritage culture labelled mental illness as “taboo” and “unreal”: *“I think it [mental health] is not really recognized in my community. People are not educated about it. They also don’t believe it’s real or a valid reason to feel some type of way”* (Participant 3, Egyptian).

Some participants also perceived their heritage cultural norms as denying that men can also suffer from mental illness. Gender role traditionalism encourages men to be dominant, stoic, and unwilling to admit to mental health problems: *“I think with the boys more than girls, they are not allowed to talk about it [mental health]. Which is negative*” (Participant 12, Kenyan).

### Cultural stigma

Participants were all cognizant of and recalled events of stigmatization in their lives. They often reported that people who suffered from mental illnesses suffered shame and stigma in their cultural community. For example, one participant from a collectivist culture felt stigmatized by her friends’ reactions to her mental illness: *“You disclose that you have a mental illness and suddenly you know they regard you differently. I heard some awful things … . When my friends found out that I had depression, they were making jokes about suicide and stuff because they thought it would be funny and this was something I really struggled with. Our culture says a lot about mental health disorders and people tend to think it is trendy”* (Participant 14, Indian). Cultural stigma associated with mental illness also influenced parents to disregard a few participants’ mental illness upon disclosure: *“ … My parents don’t think ADHD is real, so they thought I was lying to get Adderall or something which was weird*” (Participant 05, Iranian).

### Impact of cultural stigma

A recurring impact of the cultural stigma regarding mental illness was intergenerational tensions between participants and their parents. Most of the participants reported that their parents were not willing to experience mental health-related cultural stigma, so they would not entertain mental health and illness discussions with participants. This led many of the participants to live with their mental health conditions in silence with a few others wanting to seek help without the notice of their parents: *“In terms of telling my parents whether I would seek help or not, it’s something that I would probably hide from them due to stigma. They would worry and I don’t want them to. I would seek help personally knowing what they can do”* (Participant 2, Chinese Filipino).

Rather than having mental health discussions with their parents, a few other participants felt the need to talk to people outside their heritage culture for solace and to face less stigma. In addition, because men who open up about their mental health tend to especially suffer from cultural stigma, one male participant felt that going outside his cultural heritage could be the only way forward: *“If I am to bring up mental illness and related stuff, I don’t tend to talk about it with my fellow Filipinos. I will talk about it with people from other cultures who are open towards it”* (Participant 6, Filipino).

While participants’ parents often believed that mental illness does not exist, the participants themselves recognized its existence, therefore revealing intergenerational tensions. This tension was dominant among Iranians, Indians and Filipinos: *“ … My parents don’t really believe in ADHD which is weird because I literally have it. I had the diagnosis, I’m not just saying this out of nowhere. They thought I was faking it but I’m like why must someone fake their mental issues?”* (Participant 5, Iranian).

Some participants’ fear of being stigmatized by their traditional community influenced them to not seek professional help at all: *“In my cultural community, I still believe it is frowned upon very severely. People don’t like talking about it. People don’t even like to admit to having them [mental illness] even though deep down, they do know that they have it, and if you do have it, you are mostly outcasted by the community”* (Participant 11, Israel).

Two participants mentioned that attitudes towards mental health in their heritage cultures are getting better. Increasing socioeconomic status in recent years has reshaped their cultural community’s understanding of mental health and help-seeking. The social status of these participants meant that they were able to form a larger community within which they mostly welcome and are open to mental health discussions: *“In India, we have a lot of class divisions. So, since we belong to the upper middle class … , we are well-educated on this matter”* (Participant 14, Indian); *“I’m from South India and we are highly educated. We are more accepting of mental health and mental illness*” (Participant 8, Indian).

### Media, celebrity, and stigma

The role of the media and celebrities in influencing stigma emerged from the data as a trans-cultural factor that influenced all our participants, regardless of their cultural heritage. It reflected youth culture and their consumption of digital media.

First, there was a mixture of positive and negative accounts of media’s role in MHL and cultural stigma. A few participants stated that films and social media showcased the various mental illnesses and educated the public with credible mental health information. For example, some felt the media debunked the idea that mental illness does not exist: “*When we watch a TV show about mental health, they show a good point of view. Like how hard, how real, and important it is to treat it as a real issue. I think most TV shows are showing the real deal, and especially in teenage TV shows on Netflix*” (Participant 7, Brazil); *“When it comes to YouTube videos and TikTok, people try to be more positive about it [mental illness] and say get help if you need it, you’re not alone and you can get through this”* (Participant 10, Filipino).

Conversely, many of the participants criticized mental health-related films made in Canada or other Western countries for doing more harm than good. A recurring theme in the data was that participants described mental health-related films as not representing the true social, emotional, and psychological experience and impact of mental illness, but rather dramatized and exaggerated the symptoms of various mental illnesses for entertainment purposes: *“In horror movies, there’s normally a bunch of people, I don’t know if you know what I’m talking about where they say they have schizophrenia, and they make them look like they are monsters when they are not. It’s just the movie exaggerating things, trying to make people look crazy”* (Participant 10, Filipino).

Interestingly, one Indian participant added that Western mental health-related films reinforced more traditional norms regarding mental health/illness, such that men are not permitted to feel vulnerable or affirm their mental illnesses: “*Male protagonists in a movie are often told to buck up and move past it [mental illness]. This sort of promotes the idea where men are supposed to just get over themselves and move forward with their lives”* (Participant 08, Indian).

Second, our data showed that, across cultures, celebrities were also perceived to influence MHL and cultural stigma. Participants often perceived celebrities’ role as a “double-edged sword”. They reported the advantages of celebrities serving as role models and allies: because they have a large fan base, they can positively influence the masses when they openly share their mental health stories and vulnerabilities with their followers. Celebrities can let others know that they are not alone, hence creating solidarity and awareness. In light of this, some participants felt that the transparency of celebrities about their mental illness encourages them to seek professional help when the need arises: *“Thinking of celebrities, we’re like oh their lives are perfect. They have everything they need. So, seeing someone like that feeling the same things you are feeling can make you feel better about yourself and just know that you’re not alone, a lot of people experience this, even if it may seem that their lives are perfect. So, I think that’s a positive step forward on creating awareness*” (Participant 3, Egyptian); *“Take like Demi Lovato, someone who is an advocate when it comes to mental health issues. Which I think is fantastic because it’s good to see the people up there, like actually caring for people down here. We’re just scratching the surface of that stigma.”* (Participant 14, Indian).

However, one participant who was living with mental illness noted that the disparity in access to high-quality and accessible mental health services in Western cultures demonstrates a clear divide between celebrities and the people that follow them. The wealth and fame of celebrities allows them to have access to the most expensive care, whereas the average person cannot afford or gain access. Consequently, celebrities’ transparency about their excellent but not easily attainable care by the average or below-average person can lead to false hopes: *“They [celebrities] really go against the whole issue of access to mental health care resources. These people have all of the resources in the world to get help and talk about their mental health. Not all people have that access in society, and they make it look so easy. I’ve been feeling like this, it’s awful … Like rehab probably cost like $700 a day that a lot of people don’t have*” (Participant 14, Indian).

### Extinction of mental health stigma

Participants showed displeasure with the continued mental health stigma fuelled by cultural beliefs from their infancy to their current stage in life. For example, a recurring term in the data was “die out”, such that they wanted the cultural stigma regarding mental illness to become extinct. One popular recommendation to facilitate this extinction was to include mental health in the education curriculum from early childhood to enhance cultural sensitivity about MHL. This was to engage people from more ethnocultural communities to unapologetically seek help when they need it and also discredit cultural stereotypes regarding mental health: *“It [mental health] should be encouraged more like in middle school, high school and like even university and workplace. To just teach our current generation to talk about it more with their kids so that the stigma we’ve experienced from the older generation dies out”* (Participant 3, Egyptian); *“When you hear other people speaking badly about it [mental illness], I feel like people just ignore it instead of correcting them. So I feel it’s really important to correct people and it’s something I don’t really see enough in my culture. So, I feel like that’s another reason people specifically from my culture see it as such a taboo because they hear something from one person, and then they just continue with that mindset instead of correcting them*” (Participant 4, Indian).

Another recurring theme was the implementation of governmental policies and interventions to enhance the MHL of citizens; “*Governments should have active policy interventions or actively fund research in psychology so this might also make people more aware of what is going on, and really give importance to mental health the same way that the government fund research in medicine or in engineering. I think we should equally fund research in order to help dispel the stigma”* (Participant 14, Indian).

## Discussion

This qualitative study is among the few that have explored the role of culture and its influence on MHL and stigma among bicultural youths in Canada. It answers the call from other researchers who have suggested that culture plays a critical role in MHL, but there is a research gap that needs filling, especially among individuals from more collectivist heritage cultures (Altweck et al., 2015; Ryder & Chentsova-Dutton, 2012; Wang et al., 2019). Understanding these important cultural influences is critical to improving the cultural sensitivity of programmes in the future, such as university mental health services. Our study advances current understanding by demonstrating how cultural themes are embedded in bicultural youths’ comprehension, attitudes, and relational barriers to MHL and stigma alleviation. We discuss the themes that emerged in our interviews in greater detail below.

### The development of understanding of mental health

All participants were able to describe what constitutes mental health and were able to describe in detail the various facets of mental illness. This is congruent with previous studies suggesting a considerable reservoir of knowledge about mental health and illness among university students (Almanase, 2021; Furnham & Hamid, 2014). However, this may also be a result of sample selection bias—participants’ high MHL may have motivated them to participate in our study.

Nonetheless, it was evident that many of the participants had limited understanding of mental health prior to commencing their undergraduate studies. Some students attributed this to their socialization, which resulted in little or no education on mental health before starting university. Although their cultural upbringing might have reduced many of our participants’ exposure to MHL, the advent of the COVID-19 pandemic influenced at least one family to suddenly begin to engage their children in mental health discussions. Given the novelty of the COVID-19 pandemic, further research is warranted to explore its impact on bicultural youths’ mental health experiences and how collectivism impacts parent-child power dynamics during unprecedented times.

### Cultural stigma and impact

Our findings suggested that culture is a compass that people from various backgrounds rely on to differentiate between “real” mental health disorders versus unreal or irrelevant issues (Kramer et al., 2002; Samari et al., 2022). Cultural norms led many parents to mostly disregard issues brought up by participants concerning their mental health. In many cultures, mental illness is generally perceived as unpredictable and dangerous (Gopalkrishnan, 2018; Subramaniam et al., 2017; Zhuang et al., 2017). In accordance with previous research, many of our participants reported that their heritage culture labelled mental illness as a taboo and relegated mental health issues to the background (Chatmon, 2020; Islam et al., 2017; Pang et al., 2017). Collectivist cultures, which often place a high value on personal and family honour (Hampton & Sharp, 2014; Nguyen & Bornheimer, 2014), may perceive mental illness as shameful, resulting in stigmatization (Subramaniam et al., 2017).

Another finding of the present study was the tension between the disclosure of mental illness from participants and the often-invalidating response from their parents. The tension between the parent and child in regard to mental health disclosure may be rooted in the aforementioned acculturation gap (Baker et al., 2012; Crane et al., 2005; Islam et al., 2017). It was clear in our study that some parents wanted to suppress participants’ illness or to keep it hidden, avoiding the shame associated with mental illness that is internalized due to their traditional beliefs. This acculturation gap in MHL may leave participants in a vulnerable state, suffering in silence (Asvant & Malcarne, 2008; Ruiz-Casares et al., 2015). Further research is warranted on how the acculturation gap impacts bicultural youths’ mental health, their relationship with their parents and other members of the family, and how to bridge intergenerational tensions.

### Media, celebrity, and stigma

When discussing ways that MHL could be enhanced, and cultural stigma eradicated, participants often mentioned the role of media and celebrities. This role emerged as a trans-cultural factor that influenced all of our participants regardless of their cultural heritage, perhaps reflecting bicultural youth’s faster assimilation to pervasive facets of the mainstream culture (Crane et al., 2005). In addition, this theme might reflect that media and celebrities have become a universal feature in the lives of many bicultural youths (Ghazali et al., 2021).

Unlike the seemingly low impact of social media and films on MHL promotion and the reduction of stigma in our participants, the role of celebrities seemed to be supported in our study. Consistent with previous research, the disclosure of mental illness by celebrities implied close emotional solidarity, a reduction in stigma, and help-seeking promotion (Lee, 2019; Leung, 2019). Interestingly, participants seemed to have developed an imagined and self-defined intimacy with particular celebrities in the form of a parasocial relationship—a one-sided relationship where the celebrity is unaware of the devotion, interest, and emotional energy of the fan (Horton & Wohl, 1956). However, our study also showed a clear socioeconomic divide between celebrities and their followers based on the high cost of mental health services. While the role of celebrities and media on MHL has been reported by other researchers (e.g., Lee, 2019; Leung, 2019), the perspective from bicultural youth in Canada is new. Future research might investigate whether bicultural youth form parasocial relationships with celebrities from the mainstream culture or from their heritage culture, and to what extent they internalize and possibly experience conflict over their messages about mental health.

## Limitations

Our findings should be considered in light of several limitations. First, our sample was limited by the underrepresentation of men and first-generation immigrants. Second, we may have over-sampled students with high MHL given our inclusion criteria. Third, we collected the data when learning at our university was still remote due to the COVID-19 pandemic, which may have had a particularly aversive impact on students’ mental health. Findings might differ now that most learning is in person again. Future studies might also examine bicultural individuals’ MHL in other receiving cultures—for example, collectivist cultures or countries with privatized, and often inaccessible, healthcare systems such as the USA. This could aid in understanding a broader range of perspectives and experiences among bicultural youth and tailor the development of interventions that are more effective within diverse cultures.

## Implications and conclusions

Our participants seemed to be reconciling traditional norms about mental illness from their heritage culture with MHL norms from mainstream Canadian culture. Whether MHL should be included in the educational curriculum from an early age to prepare children from all backgrounds about mental health and mental illness is up to educators and policy-makers. However, many of our participants saw the need and role of governments in their heritage countries to adequately support MHL programmes and the human services sector with operationalizable policies and monetary investments. Moreover, some of our participants felt it is incumbent on people who work in the media and entertainment industries to ensure that they are depicting accurate representations of mental illness as it may have an impact on the public. We encourage the continued involvement of celebrities in anti-stigma initiatives by openly speaking about their own mental health journeys, which may promote help-seeking behaviours. For example, celebrities might collaborate with universities—especially if they are an alumni to use their influence to raise donations for university mental health programmes while advocating for greater mental health awareness.

Overall, the present study revealed a strong understanding of mental health among bicultural university students and a recognition that culture influences MHL and stigma. As MHL becomes more widespread, bicultural university students might be more likely to seek help sooner rather than later. Celebrities may also promote MHL and help-seeking behaviours. Together, this may help bicultural youths to feel less torn between two worlds when it comes to MHL and stigma, resolving the more traditional views of their parents with the views of the mainstream culture. Our results suggest that the acculturation of MHL and stigma, and associated intergenerational differences, needs to be considered by university wellness services to enhance uptake by all members of the student body.
